# The Neuropsychology of Feature Binding and Conscious Perception

**DOI:** 10.3389/fpsyg.2018.02606

**Published:** 2018-12-21

**Authors:** Barbara Treccani

**Affiliations:** Department of Psychology and Cognitive Science, University of Trento, Trento, Italy

**Keywords:** consciousness, spatial coding, feature integration, binding, neuropsychology, unilateral neglect, Balint's syndrome, blindsight

No possible discussion about consciousness and neuropsychology can be made without acknowledging the contribution of the recently deceased, worldwide known cognitive psychologist Anne Treisman. Her renowned feature integration theory (FIT; Treisman and Gelade, 1980) has inspired a huge number of studies about the relationship between attentional processes, perception, and consciousness, both in conditions where such processes were intact and in conditions where they were impaired following brain damage. Actually, this theory has also shown some critical limitations (Humphreys, 2016). My aim in this paper is to highlight that, despite such limitations, FIT may still be a powerful interpretative framework for major phenomena related to loss of conscious perception in brain-damaged patients. In particular, I will argue that the core mechanisms of this theory (i.e., spatial attention, object spatial coding and feature binding) are critically involved in visual conscious experience. Neuropsychological evidence challenging such an involvement may just actually contribute to understand better the role of these mechanisms in conscious perception.

According to the original version of FIT, individual (basic) features of an object can be processed pre-attentively and independent of their location, but they are bound together by means of spatial attention, thanks to the fact that they occupy the same location. For example, the blue color and the orientation of the contours defining the shape of a triangular geometric figure presented in a given spatial position are first processed separately but, once this spatial position is selected by attention, these individual features are integrated to form an unitary object (i.e., a blue triangle is perceived). Individual object features can be processed implicitly, but only bound object features can access consciousness.

This is consistent with the commonly held idea that a special relation exists between space and consciousness (Campbell, 2002). Undeniably, while we can imagine, e.g., colorless objects, it is hard to represent to ourselves spaceless objects. Contents of consciousness would be inherently and necessarily “situated” (Searle, 1992): it would not be logically possible to know consciously an object without perceiving it as occupying a place in which it exists.

Such an idea implies that space is a prerequisite and predecessor of conscious awareness: the encoding of stimulus location would be a necessary condition for it to enter consciousness (cf., Driver and Vuilleumier, 2001). Aside from being intuitively compelling, this notion is also plausible from an evolutionary point of view. Indeed, spatial features are among the first characteristics of objects to be discriminated both from an ontogenetic and phylogenetic perspective (cf., e.g., Xu, 1999). Moreover, this idea appears to be fully consistent with a number of neuropsychological reports concerning the effect of human brain lesions on conscious processing.

Lesions of the human brain can produce relatively isolated visual deficits, which may or may not be accompanied by an impairment of stimulus awareness. Data show some clear consistencies that, since the original formulation of FIT, have been seen as fitting nicely the idea of a special role of spatial coding and feature binding in stimulus conscious perception (cf., Robertson, 2003). Several neuropsychological conditions have been indeed interpreted based on such an idea and in the context of this theory (see Table 1 for a schematic overview).

**Table 1:** Schematic overview of how, according to the original version of FIT (Treisman and Gelade, 1980), dissociations between the processing of spatial and content (non-spatial) features of visual stimuli observed in some (emblematic) neuropsychological conditions may account for the loss of conscious perception of these stimuli.

**Neuropsychological syndrome/symptom**	**Content features processing**	**Spatial features processing**	**Feature binding**	**Conscious perception**
**Disorders of visual gnosis limited to one specific content feature**	All except one (e.g., color)			
e.g., Achromatopsia	+	+	+	+
**Cortical blindness**				
Blindsight	−	+	−	−
**Balint's syndrome**				
Simultanagnosia	+	−	−	−
Illusory conjunctions	+	−	×	+
**Unilateral neglect**				
Contralesional omissions	+	−	−	−
Allochiria	+	×	+	+

*Following FIT, stimulus awareness, or the lack of it, observed in these conditions may be traced back to whether these features can be bound together to form integrated percepts (cf., e.g., Làdavas et al., 2000; Deouell, 2002; Robertson, 2003, 2004). See text for a description of recent findings (e.g., evidence of implicit content feature processing in blindsight patients) that question such a view. +, unimpaired (proper); −, impaired (absent); ×, impaired (improper)*.

The visual deficit caused by a brain lesion can involve a specific class of stimulus attributes or be even limited to one attribute, which can be a spatial (e.g., stimulus location) or content feature of the stimulus, and concern the shape of the stimulus or its surface properties (e.g., stimulus color). Cerebral achromatopsia (i.e., acquired color blindness caused by localized brain damage) is one of the most cited examples of the category of disorders in which the deficit involves only the processing of one surface feature. Following FIT, binding of features other than color can occur, given that all the other (spatial and content) features of the object are correctly analyzed, and spatial attention can be directed toward the object location. Accordingly, not only are the patients aware of the presence of the object, but they are also aware of all its proprieties (shape, fine details, depth, etc.), with the exception of color.

Different is the case when the impairment involves the processing of stimulus spatial features. According to FIT, indeed, losing the ability to represent the location of an object would also involve the loss of consciousness of other properties of this object, and possibly of the object itself, being the spatial representation of objects the medium for binding their features. Friedman-Hill et al. (1995) described data from a patient (R.M.) with bilateral parietal-occipital lesions and a diagnosis of Balint's syndrome, who has been often presented as one of the most severe examples of loss of space perception observed in neuropsychology (Robertson, 2012). R.M., as other Balint patients, showed simultanagnosia, that is, lack of awareness of visual objects, except for one object at a time. Furthermore, he frequently combined features of different objects into the reported one (e.g., when presented with a yellow square and a blue triangle, he might report to see a yellow triangle). He was not able to report where objects were located, even when he stared at them, whereas he showed relatively intact content feature processing. Search for a target defined by one single visual feature was somewhat spared, whereas he found it very difficult to search for the conjunction of two visual features (Robertson et al., 1997). These findings are all consistent with FIT and can be accounted for by R.M.'s inadequate spatial representation of visual stimuli. Without a correct spatial representation of an object's position, allocation of attention to this position would not be possible, and accurate binding would also be hampered. Unbound features cannot be consciously perceived. Yet, in these conditions, object features might be bound incorrectly, resulting in misconjunctions that, albeit not being veridical but illusory, can access consciousness.

A similar reasoning has been used to account for another neuropsychological syndrome, which frequently follows right hemisphere parietal damage, that is, unilateral neglect (UN). In this case too, there would be spatial loss, which, however, would be limited to one side of space (Robertson, 2004).

UN is characterized by the patient's failure to orient attention toward the side of space contralateral to the lesion (Cubelli, 2017). According to one of the most known accounts of UN, this attentional deficit would precisely result from a defective spatial representation of the contralesional hemispace (Bisiach, 1993): attention would not be orientated toward locations that patients are not able to represent. Although UN patients are often unware of contralesional stimuli, several patients have now been documented who show to be able to process implicitly the color, shape, identity, and even meaning of symbols, words, and pictures presented in the affected hemispace (e.g., Làdavas et al., 1993). To account for this surprising dissociation, it has been proposed that the impaired spatial representation of contralesional stimuli is what prevent the other stimulus features, which would be fully and adequately processed, from entering consciousness (Berti and Rizzolatti, 1992; Berti et al., 2015). Consistent with FIT, the lack of spatial coding of contralesional objects would prevent attention from being oriented toward them, thus also preventing the binding of their features and their access to consciousness (Deouell, 2002).

Some UN patients show a phenomenon known as allochiria^1^ (i.e., the tendency to perceive stimuli presented on the contralesional side of the body or space as on the ipsilesional side), which would also be in line with this view: allochiria would suggest that when a spatial code, albeit inaccurate, is attributed to contralesional stimuli (i.e., they are coded as presented on the ipsilesional side), their features can be bound together and they can enter consciousness. Provided that the representation of constituent features is intact, spatial coding would enable conscious perception (cf., Làdavas et al., 2000; Deouell, 2002).

Yet, mere stimulus spatial coding, without constituent feature processing, would not result in stimulus awareness according to FIT. Indeed, just as it is difficult to represent objects without space, it is hard to imagine conscious experiences of “locations without content” (Paillard et al., 1983). It is precisely from this perspective that some authors have interpreted the lack of stimulus awareness shown by patients with damage to the primary visual cortex (V1) who prove to be able to localize visual stimuli that they deny seeing (i.e., the so-called phenomenon of blindsight; Cowey, 2010). Blindsight in cortically blind patients and implicit processing in UN patients have been often compared with each other, and described as diametrically opposed phenomena (e.g., Làdavas et al., 2000). The former would result from the relatively intact functioning of the spatial coding system, in the face of a severe impairment of the system that analyzes object constituent features, whereas the latter would result from the opposite dissociation. In both cases, no conscious awareness of the stimulus would emerge because only bound objects can be consciously perceived: in UN, the spatial deficit would prevent feature binding, whereas, in the case of blindsight, there would be nothing to bind.

FIT has provided a useful framework within which to interpret the dissociations between impaired and preserved cognitive processing of different stimulus features observed in many neuropsychological syndromes and their relation with conscious perception. In the 39 years since the original formulation of this theory, however, data from both neurologically intact participants and brain-damaged patients have been produced that show important limitations of FIT (cf., Humphreys, 2016). In particular, several neuropsychological patients have been described who proved to be able to process implicitly much more, and more complex, stimulus properties than previously thought, making it clear that lack of stimulus visual awareness cannot be traced back to lack of processing of one specific aspect of visual stimuli.

For example, some cortically blind patients have been shown to be able, not only to localize implicitly visual stimuli presented in the blind part of the visual field, but also to *discriminate* such stimuli according to either form or surface features (Cowey, 2010). For these patients too, therefore, locations might have a content. Nevertheless, patients might remain unaware of the stimuli they are able to discriminate.

Likewise (and conversely), both Balint and UN patients have been shown to process implicitly many different spatial properties of stimuli. Indeed, spared implicit spatial processing in these patients, in spite of the severe explicit spatial deficit, can be simply inferred from the previously mentioned effects on performance of complex stimuli, such as words and pictures, of which patients are not aware. In order for the identity or meaning of such stimuli to affect performance, the spatial relations between lines, angles and other elements defining their shapes must be necessarily analyzed. Some evidence of spatial coding in the neglected hemispace of UN patients also comes from allochiria. Misallocations of contralesional stimuli in allochiria usually occur to homologous locations on the ipsilesional side (Bisiach, 1993), which suggests that the stimulus position within the contralesional hemispace is accurately represented. These findings, however, can still be accounted for by both FIT and the spatial-deficit accounts of UN and Balint syndromes: there is growing evidence that the brain utilizes multiple spatial maps (Bisiach and Vallar, 2000) and it has been proposed that parietal damage (whether it is bilateral, as in Balint syndrome, or unilateral, as in UN) does not result in the impairment of spatial maps sub-serving the analysis and representation of the structure of objects, or the position of the stimulus with reference to other objects laying in the same hemispace (cf., Robertson, 2004). Such maps may depend on the activity of spared areas of the brain and work outside awareness. In contrast, the spatial deficit in these syndromes may specifically involve spatial coordinates that relies on the viewer position (e.g, the attribution of a “left” or “right” code to stimuli with reference to ego-centric spatial axes, such as the body midline), which would be the master reference frames for the guidance of attention, and, consequently, underlie feature binding processes that can bring objects to awareness (see Robertson, 2004, for the idea that both space-based and object-based attention can be trace back to a single system of hierarchical spatial frames centered on the viewer's body and its parts).

Yet, Balint and UN patients have also been shown to process implicitly the location of stimuli according to these very ego-centered spatial reference frames (Robertson, 2004). For example, Treccani et al. (2012) tested a UN patient with a unilateral flanker task and found that, even though the patient was unaware of the stimulus flanking the central target, reaction times to the target color were influenced by both the color of the flanker and its left vs. right position with respect to the patient's body midline. Similar results have been obtained with Balint patients (cf., Robertson, 2004, 2012). Such findings clearly show that spatial coding of visual stimuli is not sufficient to let them enter consciousness even when content features of these stimuli are properly processed: the spatial, as well as non-spatial, features of stimuli can be processed and, still, these stimuli may remain at an unconscious level (e.g., Treccani et al., 2012). Therefore, the lack of awareness of a stimulus whose spatial structure and content features are adequately encoded cannot be simply traced back to the lack of the representation of its position (cf., Berti and Rizzolatti, 1992; Deouell, 2002).

These findings, albeit being inconsistent with spatial-deficit accounts of UN and Balint syndromes, are reconcilable with FIT. Indeed, in the light of this evidence, it has been proposed that the lack of stimulus awareness after brain damage may depend not so much on inadequate feature binding resulting from the impairment of either spatial or nonspatial feature processing, but on a deficit of the binding process itself (Treccani et al., 2012; see also, e.g., Van Vleet and DeGutis, 2013).

However, what is also clear from the available evidence of implicit processing in brain-damage patients is that some types of feature binding can occur without awareness and without the results of the binding process entering consciousness at all. In particular, implicit binding in the form domain (i.e., binding processes underlying the representation of visual shapes) has been shown to occur after either bilateral or unilateral parietal damage (i.e., in Balint or UN patients; Humphreys, 2016) and in cortically blind patients (e.g., Trevethan et al., 2007; Celeghin et al., 2015): even though patients may remain unware of the presented stimuli (e.g., stimuli presented in the neglected or blind field) they may show intact ability of integrating objects' parts into wholes and binding visual primitives (i.e., lines and angles) defining object shapes, as well as completing figures which partially fall within the affected part of the visual field and grouping elements belonging to the same perceptual unit according to Gestalt principles of perceptual organization (e.g., proximity, similarity).

The occurrence of implicit feature binding in brain-damaged patients may be seen as the final blow to the idea of feature binding as the underlying mechanism of object conscious experience, and may lead to the conclusion that binding does not play a major role (or, even, any role at all) in conscious perception. Yet, accumulating evidence strongly suggests that there is not a single feature binding mechanism, as originally proposed by FIT, but several mechanisms, which may vary in their dependence on both attention and conscious processing (Humphreys, 2016).

Indeed, in the most current versions of FIT (Treisman, 2006), attention is not anymore supposed to be the mechanism of binding *per se*, and it is not thought to be necessary for binding to occur either (see also Robertson, 2012; Humphreys, 2016). Pre-attentive (bottom-up) binding of features can occur thanks to processes (i.e., coding of features conjunctions in single neurons and synchronized firing of separate neurons coding different features) that take place in cortical regions involved in early steps of visual analysis (as early as V1; e.g., Seymour et al., 2010). Such types of bindings might therefore not be consciously processed. Attention-related (top-down) activation, from posterior parietal cortex, may instead have a crucial role in selecting (Treisman, 2006; Robertson, 2012) or confirming (Humphreys, 2016) certain bottom-up bindings, which would then be enabled to enter consciousness.

In particular, attention and conscious processing might not be critically involved in binding content features that have learned, rather than arbitrary, relationships (i.e., they have usually experienced together) or that share local Gestalt properties (cf., Humphreys, 2016). In these cases, correct bottom-up bindings may take place without the intervention of attention (learned feature conjunctions and Gestalt cues can guide the binding process; Humphreys, 2016) and without them entering consciousness. This would indeed account for the intact implicit binding processes underlying shape representation (e.g., binding of objects' parts) observed in UN, Balint and cortically blind patients. In contrast, attention might be crucial in integrating content features from different domains (e.g., shapes with surface features such as colors), especially when feature pairings are arbitrary and correct feature binding cannot be based on stored knowledge (Humphreys, 2016). In this case, critical confirmation of bottom-up bindings from attentive processes is needed. Most researchers in this field agree that this also provides for (i.e., requires or results in) an explicit representation of space: there is no evidence of this kind of (different-domain) bindings when there is no awareness of the position from which the to-be-bound features come from (Robertson, 2004, 2012; Humphreys, 2016).

Indeed, space might play a major role in the confirmation process: the features of an object have to be bound to its location in order to verify which features or combinations of features are presented in that location. Accordingly, I propose that it is specifically this kind of content-feature-to-location binding that requires the direct intervention of attention and that enables stimulus awareness. Spatial representations of objects (i.e., the generation of spatial codes pointing to the positions where objects have been presented) and the representation of their content features may not be sufficient to bring them into awareness, however space provides the medium for the action of attention, which, binding content features to their location, would allow conjunctions of these features to be confirmed and to be consciously experienced. Such an account would reconcile the findings of intact *implicit* spatial and binding processes in brain-damaged patients with the similarly compelling evidence of a major involvement of spatial and binding processing in conscious perception (cf., Robertson, 2004, 2012).

Consistent with the idea of a critical role of attention in binding spatial and content features, in the (previously mentioned) unilateral flanker task administered by Treccani et al. (2012), additive, rather than interactive, effects of flanker color and position were observed when the flanker was presented in the contralesional, neglected hemispace, contrary to what observed both in the patient's attended hemispace and with normal perceivers (Treccani et al., 2009). As shown by previous studies, the interaction between the effects of two flanker features critically depends on the fact that such features are perceived as belonging to the same object. These findings thus suggest that, without attention, spatial and non-spatial attributes of an object can be both coded, but as separate features (i.e., as they were conveyed by two different objects): an object's spatial code might be generated, but it would not be bound to the content features of the object to which it refers.

This idea is also consistent with other (above-mentioned) phenomena related to attentional deficits that may follow parietal damage: when content features are not tightly bound to the representation of their locations, because of a damage to the attentional mechanisms subtending this binding process, false conjunctions of features of different objects (e.g., in Balint patients) or allochiric misallocation of objects toward the focus of attention (e.g., in UN patients) can occur. Accordingly, an increased number of allochiric misallocations in UN patients has been observed when the availability of patients' attentional resources were further reduced by increasing the attentional load, that is, under dual-task (vs. single-task) conditions (Bonato and Cutini, 2016).

In conclusion, the lesson so far from neuropsychology is that, even though stimulus awareness, spatial coding and feature binding might not be connected by the close causal link originally posited by FIT, binding plays an important role in conscious experience. Consciousness does not seem to result necessarily from either stimulus spatial coding, even when it occurs along with a proper processing of stimulus content features (Treccani et al., 2012), or integration of stimulus features in the form domain (Humphreys, 2016). However, there is more than one cue that some types of feature bindings, in particular the binding of content features of objects to their location in order to form integrated and “situated” percepts, might be necessary conditions for conscious perception to occur. Neuropsychological research has still plenty to say in this regard and may help to clarify whether or not content-feature-to-location binding is sufficient to bring objects to awareness, that is, whether it really is the key mechanism that triggers conscious perception.

## Author Contributions

The author confirms being the sole contributor of this work and has approved it for publication.

### Conflict of Interest Statement

The author declares that the research was conducted in the absence of any commercial or financial relationships that could be construed as a potential conflict of interest.

## Footnotes

^1^Allochiria is usually observed in association e with unilateral neglect (UN), and it is indeed prominent in some UN patients (Robertson, 2004). However, it has also been described in patients who do not show any signs of UN (Venneri et al., 2012).
